# Nitrocellulose as a Polymeric Energetic Material: Multiscale Decomposition Kinetics, Stabilization Strategies, and Micro-Ignition Architectures

**DOI:** 10.3390/polym18161978

**Published:** 2026-08-14

**Authors:** Zhanerke Yelemessova

**Affiliations:** Institute of Combustion Problems, 172 Bogenbay Batyr Street, Almaty 050012, Kazakhstan; zh.yelemessova@gmail.com

**Keywords:** nitrocellulose energetic polymers, thin-film energetic materials, thermal decomposition and kinetics, isoconversional analysis, distributed activation energy model, zeolite stabilizers, nanothermite MICs, semiconductor bridge micro-igniters

## Abstract

Nitrocellulose (NC) is a long-established energetic polymer whose autocatalytic NO_x_-driven degradation and conversion-dependent decomposition kinetics remain challenges for thin-film micro-ignition systems. This review examines NC as an energetic polymeric binder and film-forming matrix in selected nanothermite-containing formulations and micro-igniter architectures. It evaluates reported effects of formulation composition, stabilizers, nanothermite additives, deposition route, film architecture, and device geometry on thermal behavior, ignition response, safety, and storage-related limitations. Nitrogen content, molecular-weight information, crystallinity, and morphology are treated as formulation-specific characterization data rather than universal predictors of nanothermite performance. The review critically assesses Kissinger, Ozawa–Flynn–Wall, Kissinger–Akahira–Sunose, Friedman, advanced Vyazovkin, and distributed activation-energy-model approaches. Single-step kinetic models are generally inadequate for multistep, autocatalytic NC decomposition, whereas isoconversional methods provide more informative apparent activation-energy profiles when applied appropriately. Kinetic parameters obtained using different methods, sample forms, and heating programs should not be directly compared or used alone to predict ignition behavior. The review discusses stabilizers and NC-containing MICs, emphasizing formulation-specific thermal, ignition, processing, safety, and aging outcomes. In nanothermites, NC is usually a minor component functioning mainly as a binder, dispersing matrix, film-forming material, and ignition-coupling component.

## 1. Introduction

### Nitrocellulose as a Polymeric Energetic Material: Scope and Rationale

Nitrocellulose (NC) is an archetypical polymeric energetic material, combining the structural versatility of a cellulose-derived polymer with the thermochemical reactivity of densely packed nitrate ester groups [1,2,3]. Unlike low-molecular-weight explosives such as RDX or PETN, NC possesses a macromolecular architecture that simultaneously serves as a mechanical binder [4], a film-forming matrix, and an energetic phase—a combination that has sustained its relevance from nineteenth-century propellant technology to contemporary on-chip micro-ignition systems [5,6,7]. Its β-1,4-glucopyranose backbone can contain up to three O–NO_2_ substituents per anhydroglucose unit (AGU) [8]. Its thermal behavior depends on composition, additive chemistry, processing history, and test conditions; the relative role of individual structural descriptors in NC–MIC systems remains insufficiently resolved.

Over the past decade, the scope of NC research has expanded substantially beyond classical propellant science. The emergence of nanoenergetic materials—particularly metastable intermolecular composites (MICs) based on intimately mixed nanoscale metal/metal–oxide fuel (oxidizer pairs)—has reinvigorated interest in NC as a polymeric energetic binder and matrix that binds, disperses, and co-energizes thermite components in thin-film architectures [9,10,11,12,13]. In typical Al/oxide nanothermites, NC is present as a minority phase (often below 10 wt%), but its role in solution processing, film formation, and MEMS-compatible deposition is disproportionately important for device integration and ignition, even though the inorganic thermite components dominate the mass and energy balance.

Nevertheless, the same structural features that make NC attractive impose severe constraints. Thermal instability arising from weak N–O bonds; autocatalytic NO_x_-driven degradation pathways; and sensitivity to residual acidity, moisture, and mechanical stimuli create persistent challenges for storage, aging, and device reliability. Furthermore, the kinetic complexity of NC decomposition—involving multi-step, conversion-dependent activation energies that cannot be captured by a single Arrhenius parameter—complicates both safety assessment and device-level performance prediction [14,15,16,17]. Table 1 presents a comparison of the properties of NC and the main alternative energetic polymers relevant to MEMS ignition applications.

This combination of solution processability, film-forming ability, and high energetic content has sustained NC’s relevance from nineteenth-century propellants to contemporary on-chip micro-ignition systems. Yet its lower decomposition onset temperature and autocatalytic NO_x_-driven degradation distinguish it sharply from emerging nitrogen-rich polymers such as GAP and polyGLYN. Table 1 compares NC with the principal alternative energetic polymers relevant to MEMS ignition, emphasizing that NC’s dominance is driven not by thermochemical superiority but by unmatched processability and CMOS compatibility. This trade-off between processability and thermal robustness defines the central design tension in advanced MEMS energetics and motivates the stabilizer and kinetic modulation strategies reviewed in Section 4 and Section 5.

Beyond simple property comparison, Table 1 clarifies why NC remains the dominant polymer in thin-film energetic systems despite the emergence of thermally more robust alternatives. GAP, polyNIMMO, and polyGLYN offer attractive energetic or thermal characteristics, but their dependence on crosslinking and more limited solution processability complicate direct translation into lithographically patterned or electrosprayed micro-igniter architectures. In contrast, NC combines adequate energetic output with a uniquely mature processing window, which explains why current research focuses less on replacing NC outright than on mitigating its autocatalytic instability through stabilizers, formulation design, and nanothermite coupling.

This review provides a device-oriented assessment of nitrocellulose-containing energetic films, with particular emphasis on NC–nanothermite metastable intermolecular composites (MICs) and micro-ignition architectures. The focus is on experimentally reported roles of NC as a binder, dispersing matrix, film-forming component, and source of gaseous decomposition products that can affect ignition coupling. The review does not propose universal structural specifications for nitrogen content, molecular weight, crystallinity, or morphology of NC in nanothermite formulations. These descriptors are strongly dependent on the particular NC grade, source material, nitration and processing history, and characterization method. When reported in primary studies, they should be interpreted only within the corresponding formulation and test conditions.

This review prioritizes recent primary studies of NC stabilizers, NC-containing MIC formulations, and micro-ignition devices. A limited number of earlier publications are retained only where they provide foundational mechanistic or kinetic observations that have not been superseded. The term “polymeric energetic material” is used to distinguish NC from an inert binder: in a specific formulation, NC can contribute to film formation, gas generation, and ignition coupling. This review does not treat nitrogen content, molecular weight, crystallinity, or morphology as universal controls of NC decomposition or nanothermite performance. Instead, it evaluates formulation-specific evidence and identifies the lack of matched structural, thermal, aging, and ignition datasets.

The review is organized as follows. Section 2 summarizes NC chemical structure and identifies the limits of current evidence for linking molecular-weight information, crystallinity, and processing form to NC–MIC behavior. Section 3 details the primary and autocatalytic decomposition mechanisms, including the roles of NO_x_, residual acidity, and moisture. Section 4 compares isoconversional and distributed activation energy kinetic approaches and summarizes activation energy landscapes for pure and modified NC systems. Section 5 and Section 6 focus on stabilizer families and NC–nanothermite MIC formulations, respectively, emphasizing how they modulate stability–performance trade-offs. Section 7 then connects these materials-level insights to the design of SCB, EFI, and MEMS micro-ignition architectures and identifies critical gaps in aging, operando diagnostics, and statistical reliability.

## 2. Molecular and Structural Basis of Nitrocellulose

### 2.1. Chemical Structure and O–NO_2_ Functionality

**Figure 1:**
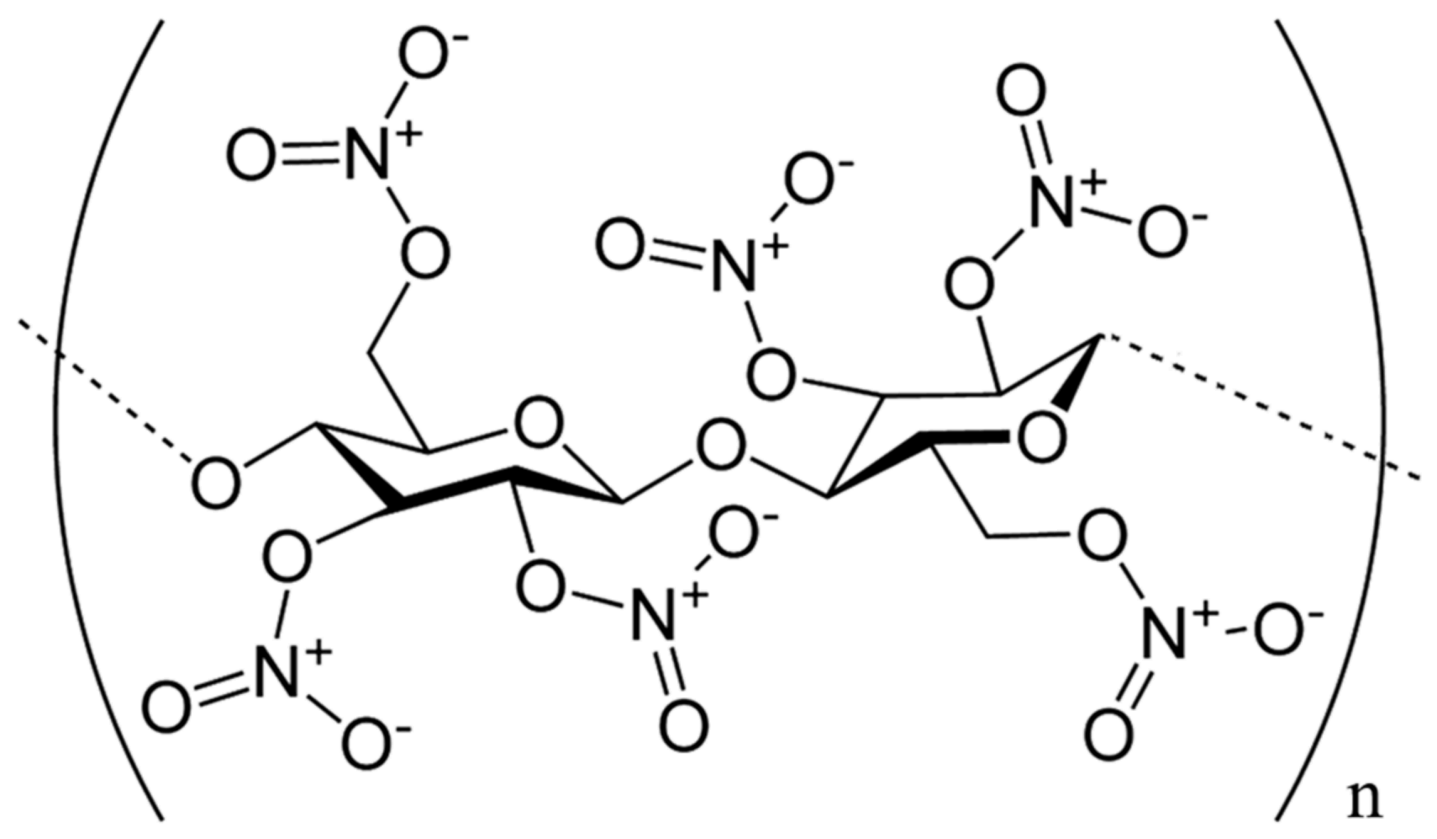
The chemical structure of fully nitrated nitrocellulose.

Nitrocellulose is produced by the esterification of cellulose with a mixture of nitric and sulfuric acids, replacing hydroxyl groups at C_2_, C_3_, and C_6_ positions of each anhydroglucose unit (AGU) with nitrate ester (–O–NO_2_) groups. The degree of substitution (DS) ranges from approximately 1.8 to 2.9 per AGU, corresponding to nitrogen content (%N) of roughly 10.7 to 13.5 wt%. The O–NO_2_ bond of the nitrate ester is substantially weaker than typical C–O bonds in ethers, with O–NO_2_ bond dissociation energies in the range of 150–175 kJ·mol^−1^ depending on the local chain conformation and hydrogen-bonding environment, as established by quantum–chemical calculations. This relatively low bond energy is the primary thermodynamic origin of NC’s energetic character and its propensity for thermal decomposition at temperatures accessible in storage and processing [18]. The chemical structure of fully nitrated nitrocellulose is presented in Figure 1 [19].

The cellulose backbone adopts a semi-crystalline structure inherited from the source biopolymer. Nitration disrupts native cellulose hydrogen-bonding networks, reducing crystallinity relative to the precursor but not eliminating it entirely. X-ray diffraction studies confirm that NC retains partial crystalline ordering, with diffraction peaks corresponding to a monoclinic unit cell whose lattice parameters depend on DS and moisture content [20,21].

### 2.2. Degree of Nitration: Thermodynamic and Kinetic Consequences

Some studies report that increasing nitrogen content within a controlled NC series is associated with altered thermal behavior; however, this relationship should not be generalized across NC grades or NC–MIC formulations. As summarized in Table 2, industrial NC grades are commonly classified by %N into collodion/lacquer, pyroxylin, smokeless-powder, and guncotton/explosive domains, each associated with characteristic processing routes and use cases.

Within these grade windows, systematic TGA/DSC studies on representative samples show that increasing %N within a given formulation series lowers the peak decomposition temperature and increases the total heat of decomposition, while shifting gaseous product distributions toward higher NO_2_ and CO fractions. Scanning Electron Microscopy (SEM) imaging consistently reveals more pronounced surface cracking and structural distortion in higher-nitrogen NC after mild heating, indicating that increased energetic group density amplifies defect formation and contributes to instability in addition to changing overall bond energetics [34,35].

### 2.3. Molecular Weight, Degree of Polymerization, and Chain Architecture

Nitrocellulose molecular weight and degree of polymerization vary with cellulose precursor, nitration conditions, stabilization, processing, and aging history. Their determination is method-dependent and can be complicated by broad or multimodal molecular-mass distributions. Solovov et al. [36] demonstrated the application of dynamic light scattering to nitrocellulose molar-mass determination after fractionation in acetone, illustrating the importance of reporting both the analytical method and the specific NC sample studied.

However, reviewed NC–nanothermite studies rarely report molecular-weight distributions together with matched thermal-decomposition or ignition measurements; this omission limits cross-study interpretation. Therefore, no universal molecular-weight or degree-of-polymerization requirement can currently be derived for NC used as a nanothermite binder. For NC–MIC studies, molecular-weight information should be reported, where available, because it may affect solution viscosity, coating quality, and film integrity. Such information should be treated as batch-specific formulation documentation rather than as an established predictor of decomposition mechanism or nanothermite performance.

### 2.4. Crystallinity: Characterization Rather than a Universal Design Rule

Nitration and subsequent processing may alter the ordered and disordered domains of cellulose-derived NC. However, crystallinity values depend strongly on the analytical method, baseline treatment, sample moisture, degree of substitution, and processing history. The currently reviewed NC–nanothermite studies do not provide sufficient matched datasets to establish a general relationship between crystallinity and decomposition mechanism, ignition delay, or storage stability. Accordingly, crystallinity should be reported when it has been measured for a specific NC–MIC formulation, but it should not be presented as a universal acceptance criterion for nanothermite applications. Future studies should combine X-ray scattering analysis with controlled thermal, aging, and ignition measurements on the same formulation.

### 2.5. Processing Form and Film Architecture

In NC-containing MIC systems, the most directly relevant morphological issue is not a universal NC morphology requirement but the architecture produced after deposition: film continuity, adhesion to the substrate, porosity, thickness uniformity, nanoparticle dispersion, and the presence of agglomerates. These properties affect practical device integration and should be measured for each formulation. Available evidence remains insufficient to define a universal morphology–decomposition relationship across NC grades, deposition routes, and nanothermite compositions. Therefore, reported morphology should be interpreted in conjunction with the specific formulation, deposition method, and ignition test.

## 3. Thermal Decomposition Mechanisms

### 3.1. Primary Initiation: O–NO_2_ Homolysis

The initiation of NC thermal decomposition is unambiguously assigned to homolytic cleavage of the O–NO_2_ bond, generating an alkoxy radical on the glucopyranose ring and a free NO_2_ radical [37]. This assignment rests on converging evidence from T-jump/FTIR spectroscopy, which detects NO_2_ as the first gaseous product at temperatures as low as 100–120 °C under fast-heating conditions; from quantum–chemical calculations that yield O–NO_2_ bond dissociation energies of 150–175 kJ·mol^−1^, consistent with the range of experimentally measured activation energies; and from isotopic labeling studies confirming that oxygen in early NO_x_ products originates from the nitrate ester and not from the cellulose backbone [38,39,40].

The alkoxy radical produced by O–NO_2_ homolysis undergoes rapid β-scission, generating carbonyl compounds and chain fragments [41]. Parallel heterolytic pathways, involving protonation of the nitrate ester oxygen by residual or product acids, also contribute under mildly acidic conditions and at temperatures below 100 °C, where they may dominate the slow aging process [42,43]. The relative contributions of homolytic and heterolytic pathways depend on temperature, local acidity, moisture content, and NC microstructure [43,44]—a complexity that accounts for some of the variability in reported activation energies across different studies and sample preparations.

### 3.2. Autocatalytic NO_x_-Driven Pathways

The most practically important feature of NC decomposition is its autocatalytic character. NO_2_ produced by O–NO_2_ homolysis rapidly equilibrates with NO and N_2_O_4_ in the gas phase. In the presence of moisture—ubiquitous in practical NC formulations—these species generate HNO_3_ and HNO_2_, which diffuse back into the polymer matrix and catalyze further denitration by lowering the activation barrier for O–NO_2_ cleavage [45,46]. This creates a positive feedback loop: each decomposition event generates acid, which accelerates subsequent events, leading to self-accelerating reaction profiles characteristic of autocatalysis.

**Figure 2:**
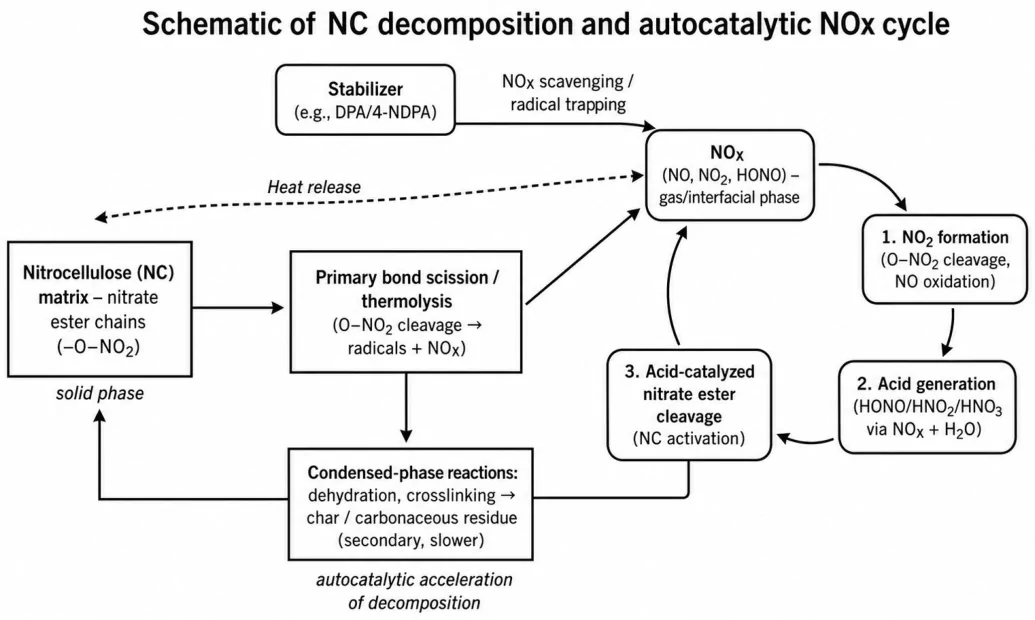
Schematic of NC decomposition and autocatalytic NO_x_ cycle.

The autocatalytic mechanism has been quantitatively described by a modified Prout–Tompkins model incorporating acid concentration as a dynamic variable [47]. Isothermal TGA data at temperatures of 90–150 °C reveal sigmoid-shaped mass-loss curves with induction periods that decrease strongly with temperature and increase with stabilizer concentration, consistent with the autocatalytic model [48]. Heat-flux calorimetry measurements confirm that the onset of rapid heat generation correlates with the accumulation of critical acid concentrations in the NC matrix, establishing a direct calorimetric signature of autocatalytic onset that can serve as a safety criterion [49]. A schematic illustration of NC decomposition pathways, highlighting the primary O–NO_2_ homolysis, autocatalytic NO_x_-driven acid formation, and the feedback loop between condensed-phase denitration and gas-phase NO_x_ evolution, is presented in Figure 2.

It is important to note that several published studies describe NC decomposition as a “single-step first-order reaction” based on model-fitting of DSC data [50,51]. This description is mechanistically inadequate for practical purposes. While single-step models may fit DSC data within a limited temperature range, they fail to capture the autocatalytic acceleration, the conversion-dependence of activation energy, and the role of diffusion limitations in dense granules—all of which are critical for accurate shelf-life prediction and MEMS device design.

### 3.3. Condensed-Phase vs. Gas-Phase Reaction Pathways

NC decomposition involves both condensed-phase and gas-phase reaction channels whose relative contributions depend strongly on temperature and heating rate [52]. At temperatures below approximately 180 °C (relevant to storage and slow aging), condensed-phase reactions—including O–NO_2_ homolysis, acid-catalyzed denitration, and ring-opening of the AGU—dominate, with NO_x_ evolution as the primary gas-phase signal [53]. At temperatures above approximately 200 °C (relevant to ignition scenarios), the decomposition of the cellulose backbone, oxidation of carbonyl intermediates, and gas-phase combustion of volatile fragments become significant contributors to total heat release [54].

TG-FTIR and TG-MS studies resolve this two-stage behavior, detecting NO_2_ and H_2_O as dominant low-temperature products and CO, CO_2_, CH_2_O (formaldehyde), and various organic fragments at higher temperatures. The transition between condensed- and gas-phase processes is influenced by heating conditions, formulation composition, confinement, and gas transport [55]. The independent effects of NC nitrogen content and morphology in NC–MIC systems require matched studies.

As summarized schematically in Figure 2, condensed-phase denitration and gas-phase NO_x_ equilibria form a coupled feedback loop whose relative importance changes with temperature, heating conditions, confinement, and gas-transport conditions.

### 3.4. Effect of Residual Acidity and Moisture

Residual nitric and sulfuric acids from the nitration process are among the most consequential impurities in NC. Insufficient washing and neutralization leave acid within mesopores and amorphous regions; at moderate storage temperatures, these directly catalyze O–NO_2_ cleavage and denitration, bypassing the induction period of the autocatalytic cycle. Heat-flux calorimetry and VST measurements demonstrate that NC with higher residual acidity (pH < 6 in the extract) exhibits shorter induction times and higher gas evolution rates at 100–130 °C, quantitatively correlating with acid content [56]. Sulfate ester residues, arising from incomplete removal of sulfuric acid, are particularly deleterious because sulfate groups catalyze ester hydrolysis with higher efficiency than nitrate-derived acids [57].

Moisture has a dual role. At low levels, moisture plasticizes the NC matrix and accelerates NO_x_–acid conversion, promoting autocatalytic onset. At very high humidity, condensed water provides a medium for acid dilution and NO_x_ absorption, which can paradoxically retard gas-phase autocatalysis while simultaneously promoting hydrolytic backbone degradation [58]. These opposing effects mean that there is no simple monotonic relationship between moisture content and NC stability—a nuance frequently overlooked in stability assessments that measure only total weight change.

### 3.5. Modern Mechanistic Models and Computational Insights

Recent advances in computational chemistry have enabled detailed modeling of O–NO_2_ bond cleavage and early decomposition pathways in NC. Density functional theory (DFT) calculations at the B3LYP/6-311G(d,p) level reproduce experimental O–NO_2_ bond dissociation energies within 5–10% and confirm that C_2_- and C_6_-substituted nitrate esters have slightly lower bond energies than C_3_-substituted groups, predicting a positional selectivity in initial decomposition that has been partially supported by isothermal NMR denitration studies [59]. ReaxFF molecular dynamics simulations of NC decomposition at high temperatures capture the multi-step character of backbone fragmentation, correctly predicting the sequence of major gas-phase products (NO_2_ → CO/CO_2_ → H_2_O → organic fragments) observed in TG-MS experiments [60]. These computational tools remain limited by system size—tractable simulations cover only tens to hundreds of AGUs and therefore cannot represent the full molecular and morphological heterogeneity of practical NC materials. Bridging this scale gap represents a major methodological opportunity, particularly for multiscale modeling frameworks that couple atomistic ReaxFF simulations to continuum-scale kinetic and transport descriptions.

While Section 3 has focused on qualitative and semi-quantitative mechanistic models, practical stability assessment and device design require these mechanisms to be expressed in kinetic terms. Section 4 therefore formalizes NC decomposition in the language of activation energies and rate equations, comparing isoconversional and DAEM approaches and highlighting their implications for service-life prediction and ignition modeling.

## 4. Decomposition Kinetics

### 4.1. Isoconversional Methods: Principles and ICTAC Recommendations

The International Confederation for Thermal Analysis and Calorimetry (ICTAC) recommends isoconversional (model-free) methods as the primary approach for kinetic analysis of thermally activated processes in polymers and composites, because they yield activation energy as a continuous function of conversion, E_a_(α), without assuming a specific reaction model [61]. The general form of the rate equation is:

(1) $$\frac{d\alpha }{dt}=A\;exp(-\frac{E_{a}}{RT})f(\alpha ),$$

where α is conversion degree (dimensionless, 0–1) defined as the fraction of total mass loss or heat release; *t* is time (s); *A* is the pre-exponential (frequency) factor (s^−1^); *E_a_* is the apparent activation energy (J·mol^−1^); *R* is the universal gas constant (8.314 J·mol^−1^·K^−1^); *T* is the absolute temperature (K); and *f*(*α*) is the differential reaction model function. For isoconversional analysis, *f*(*α*) is eliminated by evaluating Equation (1) at the same α across multiple heating rates *β*, yielding effective *E*_*a*_ profiles.

This is the linearized form of the integral kinetic equation, obtained by substituting the Doyle approximation into the temperature integral [62]. The Ozawa–Flynn–Wall (OFW) integral method [63] yields:

(2) $$\ln\beta =\left[\ln\left(\frac{AE_{a}}{Rg\left(\alpha \right)}\right)-5.331\right]-1.052\frac{E_{a}}{RT_{\alpha }},$$

where β—heating rate, A—pre-exponential multiplier, E_a_—activation energy, g(α) is the integral reaction model, T_α_—the temperature at conversion α, −5.331—the Doyle constant and −1.052—the Doyle slope coefficient.

The Kissinger–Akahira–Sunose (KAS) method [64] improves upon OFW by using a more accurate approximation:

(3) $$\ln\left(\frac{\beta }{T_{\alpha }^{2}}\right)=ln\left(\frac{AR}{E_{a}(\alpha )g_{a}(\alpha )}\right)-\frac{E_{a}(\alpha )}{RT_{\alpha }},$$

where β—heating rate; T_α_—temperature (K) at which the conversion of α is achieved at a given β; A—pre-exponential factor; E_a_(α)—activation energy at the conversion rate α.

The Friedman differential method [65] avoids integral approximations entirely:

(4) $${\ln\left(\frac{d\alpha }{dt}\right)}_{\alpha }=ln[A\;f\left(\alpha \right)]-\frac{E_{a}(\alpha )}{RT_{\alpha }},$$

where (d_α_/d_t_)_α_—reaction rate at a fixed α, A—the pre-exponential multiplier, *f*(α)—the differential function of the model, ln[A *f*(α)]—free member, E_a_(α)—activation energy, and T_α_—temperature of reaching α, providing higher sensitivity to multi-step behavior at the cost of amplified noise.

The Kissinger equation is strictly applicable only to single-step processes [66]:

(5) $$\ln\left(\frac{\beta }{T_{p}^{2}}\right)=\ln\left(\frac{AR}{E_{a}}\right)-\frac{E_{a}}{{RT}_{p}},$$

where β is heating rate, T_p_—the DSC peak temperature, A—pre-exponential factor, and E_a_—apparent activation energy. This equation is widely used for rapid comparison but should not be the sole basis for mechanistic conclusions in multi-step systems such as NC.

The advanced Vyazovkin method eliminates the linearization approximation by minimizing a functional based on the exact temperature integral, providing the most accurate E_a_(α) estimates and is strongly recommended for NC composites where multi-step behavior is common. Table 3 summarizes the main kinetic methods used to analyze the thermal decomposition of NC.

Among these approaches (Table 3), peak-based Kissinger analysis is valuable for rapid, comparative screening of different nitrocellulose formulations, but it should not be relied on alone for mechanistic interpretation of an autocatalytic, multi-step decomposition process. Integral isoconversional methods (Ozawa–Flynn–Wall, Kissinger–Akahira–Sunose) remain widely used and provide convenient access to E_a_(α) profiles; however, their accuracy depends on the chosen heating-rate range and on the validity of the integral approximations employed. In contrast, the differential Friedman method and the advanced Vyazovkin approach yield more robust activation energy trends for NC and NC-based composites. These methods require higher data quality and greater computational effort and are therefore preferable when detailed kinetic analysis or service-life prediction is the objective.

In the following sections, we therefore emphasise isoconversional and advanced Vyazovkin treatments. Kissinger plots are used primarily as auxiliary tools for benchmarking and comparing NC-based energetic formulations.

### 4.2. Distributed Activation Energy Model (DAEM)

The DAEM represents decomposition as a superposition of parallel first-order reactions with a Gaussian distribution of activation energies [67], characterized by a mean E_a_ and standard deviation σ:

(6) $$1-\alpha =\int_{0}^{\infty }\varphi \left(E\right)\exp\left(-A\int_{0}^{t}exp\left(-\frac{E}{RT}\right)dt\right)dE,$$

where *φ*(E) is the activation energy distribution function (typically Gaussian):

(7) $$\varphi \left(E\right)=\frac{1}{\sigma \sqrt{2\pi }}exp\left(-\frac{{\left(E-E_{0}\right)}^{2}}{2\sigma^{2}}\right),$$

with *E*_0_ the mean apparent activation energy (J·mol^−1^), *σ* the standard deviation of the distribution (J·mol^−1^), and A the pre-exponential factor assumed uniform across all parallel reactions. All other symbols as previously defined.

Equation (6) describes thermolysis as a superposition of an infinite number of parallel, independent first-order reactions, each characterized by its own energy barrier, E. DAEM [68] may be useful for representing broad and multi-step decomposition profiles; however, its specific mechanistic interpretation for NC remains insufficiently validated. Despite its suitability, DAEM has rarely been applied to NC systems—a significant gap identified in the literature reviewed here. The few available applications to cellulose pyrolysis confirm that DAEM captures the broad decomposition profiles more accurately than single-step models, motivating its systematic application to NC and NC–additive composites.

In practical applications, φ(E) is typically obtained by fitting DAEM parameters to non-isothermal DSC or TGA curves using numerical inversion techniques such as the Miura–Maki method or extended Kissinger analysis [69]. For cellulose pyrolysis, DAEM has been shown to reproduce broad decomposition profiles more accurately than single-step models, with σ values on the order of 20–40 kJ·mol^−1^. To date, analogous DAEM analyses for nitrocellulose are scarce, and most studies still rely on isoconversional methods with implicit single-barrier assumptions. Systematic DAEM implementation for NC and NC–additive composites therefore represents a promising route to quantifying the heterogeneity of O–NO_2_ bond environments and morphological microstructures.

### 4.3. Kinetic Compensation Effect

A persistent challenge in NC kinetic analysis is the kinetic compensation effect (KCE): across different formulations and studies, ln A correlates linearly with E_a_, making it difficult to separate genuine mechanistic differences from artifacts of model fitting and experimental protocol. The KCE is particularly pronounced when comparing results across different DSC instruments, sample masses, and crucible geometries, because heat transfer artifacts shift T_p_ and thereby bias both A and E_a_ estimates [70,71]. Awareness of the KCE is critical when comparing kinetic parameters for NC systems across the literature: differences in reported E_a_ between laboratories may partly reflect experimental protocol rather than genuine compositional effects. ICTAC recommends validating kinetic parameters by predicting independent isothermal experiments before interpreting E_a_ differences as mechanistically significant.

### 4.4. Comparative Activation Energy Landscape

Table 4 summarizes activation energies reported in the literature for NC-based systems across multiple methods and formulations, drawn from primary sources identified in this review. The table is intentionally restricted to studies that (i) report explicit activation energy values for NC-based systems, (ii) specify the kinetic method used (OFW, KAS, Friedman, advanced Vyazovkin), and (iii) provide sufficient experimental detail (sample type, %N, morphology) to allow qualitative comparison. The variation reflects differences in sample identity, formulation, thermal history, instrument configuration, sample mass, crucible geometry, heating program, and kinetic method. Many publications do not report sufficient NC characterization to isolate contributions from individual structural descriptors. Second, within the advanced Vyazovkin data set reported in [72,73], ion-exchanged zeolite stabilizers (Cu-, Ag-ZSM-5) consistently yield higher mean E_a_(α) profiles than the pure NC baseline studied by the same group, with increases on the order of 10–25 kJ·mol^−1^ across the conversion range. These values, while mechanistically plausible, derive from a single laboratory and should be regarded as indicative rather than definitive until confirmed by independent studies. Third, reported NC–nano-oxide and NC–MIC systems exhibit formulation-dependent changes in apparent Ea. Some studies report lower values within a matched formulation series, whereas other reported systems show unchanged or higher apparent E_a_. Because the kinetic method, heating program, reference formulation, and sample geometry differ among studies, no universal direction of E_a_ change can be assigned to nanothermite addition. Accordingly, the present review interprets apparent kinetic parameters only within the experimental context of the original study.

From a formulation design perspective, the ranges in Table 4 should be treated as order-of-magnitude guidance rather than fixed targets. For pure NC, E_a_ values near 150 kJ·mol^−1^ imply that a 10–20 kJ·mol^−1^ increase induced by zeolite stabilizers can, under typical AOP-48 assumptions, extend chemical life by several years at ambient storage temperatures. In some matched formulation series, nanothermite additives are associated with reduced ignition thresholds or shorter ignition delays. However, these device-level outcomes cannot be inferred from apparent E_a_ alone because they also depend on interfacial heat generation, particle dispersion, film architecture, and the ignition method. Integrating these kinetic shifts into standard life-prediction frameworks therefore offers a quantitative route to balancing stability and performance in NC-based energetic films.

### 4.5. Isoconversional Trends and Formulation-Specific Interpretation

**Figure 3:**
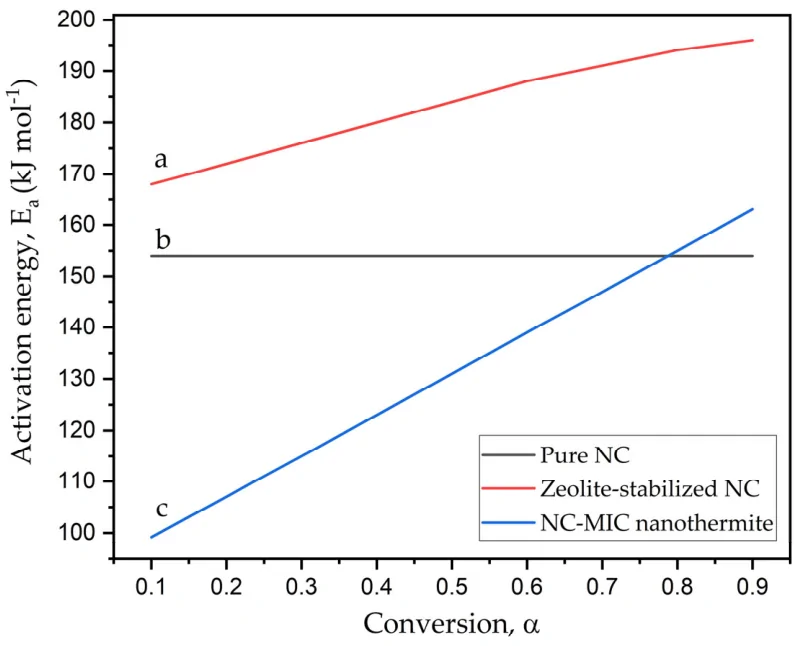
Schematic apparent activation-energy profiles, E_a_(α), illustrating trends reported for (**a**) zeolite-stabilized NC, (**b**) selected pure NC, and (**c**) NC-containing MIC formulations. The profiles are formulation-, method-, and test-condition-dependent and are presented as examples rather than universal kinetic signatures. Comparisons are meaningful only within matched experimental series; see Table 4.

Isoconversional analysis of E_a_(α) reveals characteristic signatures of different decomposition regimes. For pure NC, E_a_(α) typically decreases modestly (5–15%) as α increases from 0.1 to 0.8, consistent with a transition from an initiation-controlled regime (O–NO_2_ homolysis) to an autocatalytic regime where the effective barrier is lower. In zeolite-stabilized NC, E_a_(α) remains elevated across the full conversion range, indicating that NO_x_ adsorption by the zeolite suppresses autocatalytic acceleration throughout the decomposition process. Reported E_a_(α) profiles can help describe thermal behavior within a matched formulation series, but they should not be treated as universal signatures of specific additive classes. In particular, available data do not justify the conclusion that nanothermite additives uniformly lower apparent activation energies. Where a formulation shows a reduced ignition threshold or shorter ignition delay, this outcome may also reflect interfacial heat release, particle dispersion, film architecture, gas transport, and device-specific electrical coupling (Figure 3).

Having established how kinetic parameters respond to NC formulation and additive chemistry, the next logical step is to examine how stabilizers are designed to exploit these mechanistic and kinetic sensitivities. Section 5 thus transitions from descriptive kinetics to the targeted modulation of NC stability via classical, bio-derived, and zeolite-based stabilizers.

## 5. Stabilizers: Mechanisms and Modern Developments

### 5.1. Classical Stabilizers: DPA, Centralites, and Akardites

Diphenylamine (DPA), symmetrical and unsymmetrical centralites (diethyl centralite, dimethyl centralite), and akardites (methyl akardite, ethyl akardite) represent the classical stabilizer family used in NC-based propellants for over a century. Their stabilizing mechanism is based on scavenging of NO and NO_2_ radicals and gases: DPA reacts rapidly with NO_2_ to form nitrosodiphenylamine and then nitrodiphenylamine derivatives, consuming the autocatalytic agents before they can re-enter the polymer matrix and catalyze further denitration [82,83]. The formation of these nitro derivatives is irreversible and stoichiometric, meaning that stabilizer lifetime is finite and can be predicted from initial concentration and aging conditions, providing a framework for service-life estimation.

DSC and isoconversional kinetic studies confirm that DPA increases E_a_ by approximately 10–30 kJ·mol^−1^ and shifts T_p_ by 5–15 °C relative to unstabilized NC. The magnitude of this effect depends critically on DPA concentration, dispersion homogeneity, and aging history (i.e., how much DPA has already been consumed by NO_x_) [84]. A key limitation is that DPA and its nitro derivatives have measurable vapor pressures at elevated temperatures and can migrate out of thin NC films, a phenomenon particularly relevant to MEMS-scale devices where film-to-substrate area ratios are high.

### 5.2. Green and Bio-Derived Stabilizers

Growing environmental and toxicological concerns over DPA and centralites have stimulated research into bio-derived stabilizers. Lignin, guaiacol, curcumin, and plant polyphenols share the capacity to donate hydrogen atoms to free radicals and to react with NO_x_, acting as both radical scavengers and NO_x_ absorbers [85,86]. TGA/DSC studies on lignin-stabilized NC/DEGDN composites show reduced mass loss rates and increases in E_a_ by 10–30 kJ·mol^−1^ compared to DPA-stabilized analogues, with decomposition onset temperatures 5–12 °C higher. Recent state-of-the-art reviews on stabilizers for nitrate ester-based energetic materials underscore this point. They emphasize that mechanistic diversity (radical scavenging, NO_x_ trapping, acid neutralization) and protocol heterogeneity complicate direct performance ranking across stabilizer families. The comprehensive analysis by Trache [87] explicitly calls for harmonized aging and kinetic testing to enable quantitative comparison of classical and emerging stabilizers.

Fullerene (C_60_) and functionalized carbon nanomaterials have also been evaluated as radical-scavenging stabilizers, exploiting their large surface area and high reactivity toward peroxy- and oxy- radicals. These offer the advantage of minimal vapor pressure and therefore low migration tendency, which is attractive for thin-film device applications. However, their cost, potential agglomeration, and effects on NC film processability remain barriers to widespread adoption. Direct quantitative comparisons between bio-derived stabilizers and classical DPA are still scarce, and reported claims of superior performance often rely on non-standardized protocols that limit cross-laboratory comparability [88].

### 5.3. Zeolite-Based Porous Stabilizers: Mechanism and Performance

A particularly significant development is the application of ion-exchanged zeolites as porous stabilizers that function primarily by adsorbing NO_x_ products rather than by radical scavenging. Building on foundational studies of NO decomposition over Cu–ZSM-5 in automotive catalysis, recent work has demonstrated that ion-exchanged ZSM-5 can substantially improve NC thermal stability, with Chebbah [73] and Trache [87] showing extended MV discoloration times and reduced VST gas evolution for ZSM-5-containing NC formulations. These results position zeolite-based stabilizers alongside DPA and bio-derived phenolics as a third major stabilizer family for energetic polymers [84] (p. 213).

Mechanistically, the zeolite framework acts as a molecular sieve that captures NO and NO_2_ molecules within its micropore channels. The ZSM-5 pore window diameter of ~5.5 Å readily admits NO_2_ (kinetic diameter ~3.5 Å), enabling rapid diffusion and adsorption within the zeolite channels. This adsorption prevents NO_x_ species from re-entering the NC matrix and thereby interrupts the autocatalytic cycle primarily at its propagation stage rather than at initiation. Cu^2+^ exchange sites in ZSM-5 are known from automotive SCR catalysis to promote NO oxidation and NO_x_ decomposition, suggesting that similar pathways could operate in NC–zeolite composites. However, under NC decomposition conditions, this has not yet been confirmed directly by isotope-labeling or operando gas analysis, and current interpretations should be regarded as mechanistic hypotheses rather than established fact. Likewise, the larger pore windows of FAU and MOR zeolites imply potentially higher capacity for N_2_O_4_ adsorption than MFI-type frameworks, but comparative NC stabilization studies remain too limited and protocol-dependent to support definitive performance rankings.

**Figure 4:**
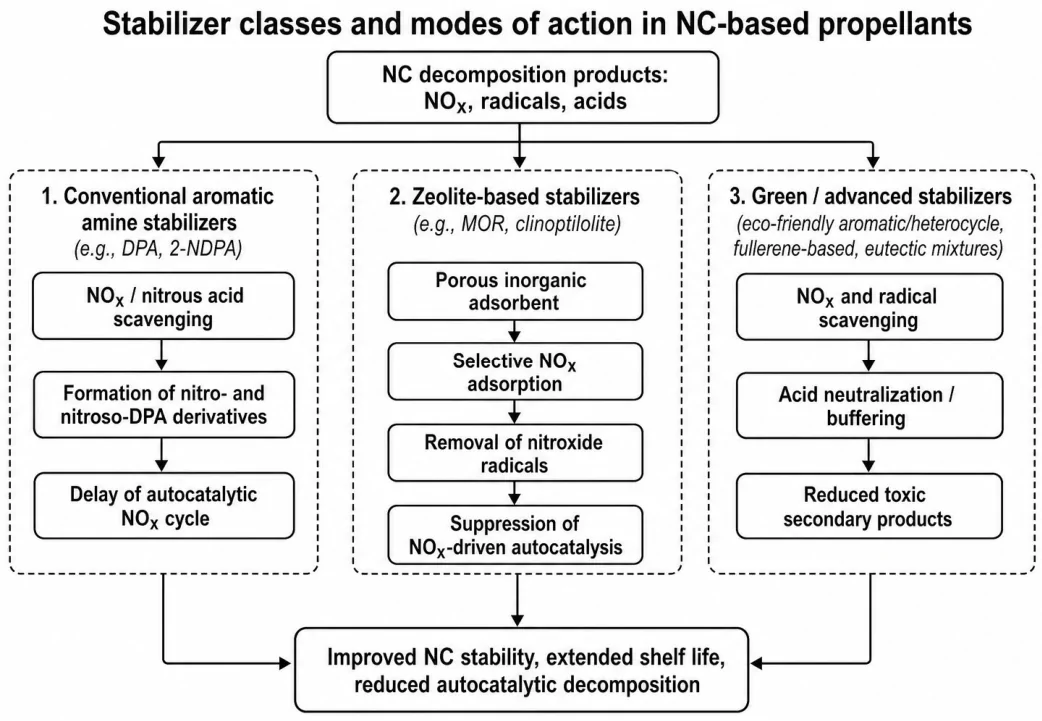
Schematic of stabilizer classes (DPA, zeolite, green stabilizers) and their modes of action.

Conceptual comparison of stabilizer mechanisms in nitrocellulose: (a) classical DPA-type stabilizers that function through stoichiometric NO_x_ scavenging; (b) bio-derived phenolic stabilizers that combine radical and NO_x_ trapping; and (c) ion-exchanged zeolite stabilizers that act as porous NO_x_ adsorbents and potential catalytic NO_x_ destructors, as illustrated in Figure 4.

Within the advanced Vyazovkin dataset reported for a single NC formulation series, Cu-ZSM-5 raises mean E_a_(α) from ~154 kJ·mol^−1^ (pure NC baseline) to approximately 174–196 kJ·mol^−1^. These increases are among the largest stabilizer-induced shifts reported in that specific study and highlight the potential of zeolite-based porous stabilizers [84] (p. 217), but broader confirmation across independent laboratories is still needed [89,90]. Given that these values originate from a single laboratory, employ a particular kinetic protocol, and are potentially sensitive to noise and sample preparation, they should be interpreted as evidence of outstanding performance in reported studies rather than as a definitive demonstration of global superiority over all stabilizer classes. Interlaboratory validation using standardized isoconversional protocols is necessary before Cu–ZSM-5 or related zeolites can be regarded as universally optimal NC stabilizers.

The implications for on-chip devices are significant: zeolites can be incorporated into NC thin-film formulations as nanoparticulate additives without substantially altering the film-forming and solubility characteristics of NC, potentially providing long-term stability in MEMS-integrated energetic layers. However, the long-term performance of zeolite-stabilized NC films under thermal cycling, humidity, and mechanical stress—conditions representative of actual device storage and use—has not yet been systematically studied.

The zeolite stabilizer family extends beyond ZSM-5 (MFI topology). Mordenite (MOR), with its one-dimensional 12-membered ring channels (pore diameter ~6.5–7.0 Å), scavenges nitroxide radicals via a distinct diffusion-limited mechanism compared to ZSM-5 [83] (p. 114). Faujasite (FAU) zeolite, with pore windows of 7.4 Å sufficient to accommodate N_2_O_4_ dimers (~6.4 Å kinetic diameter), is predicted to have higher capacity for autocatalytic NO_x_ species than MFI-type zeolites based on pore size considerations, although this hypothesis has not yet been tested directly in NC stabilizer design [84] (p. 219). Under the specific VST and Bergmann–Junk protocols employed, MOR, BEA, MFI, and FAU zeolites exhibit stabilizing efficiency that equals or exceeds DPA for the tested NC formulations. However, the limited number of comparative studies and variations in test conditions preclude a definitive performance ranking between zeolites and classical aromatic amines.

### 5.4. Stabilizer Degradation Kinetics and Service Life Prediction

Classical stability tests (VST, Methyl Violet, Bergmann–Junk) provide pass/fail criteria but do not yield quantitative service-life predictions. Modern approaches based on accelerated aging combined with isoconversional kinetic modeling can extrapolate stability parameters to storage conditions [91]. The stabilizer consumption model [51,92] treats diphenylamine (DPA) (or equivalent stabilizer) concentration C(t) as a function of time under isothermal conditions according to:

(8) $$\frac{dC}{dt}=-k(T)C^{m}{\left[{NO}_{x}\right]}^{n},$$

where *C*—stabilizer concentration, *dC*/*dt*—stabilizer degradation rate, *k*(*T*) is an Arrhenius rate constant, m—partial stabilizer reaction order (usually m = 1), [NO_x_]—the local concentration of autocatalytic gases, and *n*—partial reaction order with respect to NO_x_.

In practical aging studies, the partial reaction orders m and n in Equation (8) are determined by combining isothermal aging at several stabilizer concentrations with independent measurements of NO_x_ or acid concentration. For classical DPA-stabilized NC, m is typically close to unity (first-order in stabilizer), while n is inferred from the temperature dependence of induction times and gas evolution, often yielding an apparent order between 0.5 and 1 with respect to autocatalytic species. Experimental protocols use UHPLC or GC to quantify residual stabilizer and acid content as a function of time, followed by nonlinear regression of Equation (8) to extract k(T), m, and n under controlled humidity and temperature.

For zeolite-based stabilizers, where NO_x_ capture is non-stoichiometric and governed by adsorption equilibria rather than direct chemical consumption, an equivalent life prediction model can be constructed by replacing [NO_x_] in Equation (8) with an effective free NO_x_ concentration derived from a Langmuir or Temkin adsorption isotherm and introducing a saturation capacity term for the zeolite. In such a framework, the time to reach a critical free NO_x_ level—corresponding to the onset of rapid autocatalysis—becomes the relevant service-life metric, and both adsorption equilibrium constants and zeolite capacity must be measured under NC matrix conditions. While this approach has not yet been fully implemented for NC–zeolite systems, it offers a pathway for extending established stabilizer depletion models to non-stoichiometric porous stabilizers.

A critical methodological limitation of classical bulk thermal methods (DSC, TGA, VST) is their inability to resolve spatial heterogeneity in stabilizer depletion. A study [93] demonstrated that Ultra-High-Performance Liquid Chromatography (UHPLC) analysis of NC-based propellants aged under hot and cold conditions detected localized decreases in nitroglycerin and stabilizer content varying from the liner surface to the interior—variations that DSC and heat-flow calorimetry failed to resolve entirely [93]. For MEMS-integrated NC thin films, where energetic layer thickness is 5–20 µm and spatial uniformity directly governs ignition reliability, UHPLC or analogous micro-scale chemical imaging techniques represent a critical unmet analytical need.

This formulation captures the depletion kinetics observed in long-term (months to years) aging experiments and can be coupled to polymer degradation models to predict the onset of dangerous autocatalysis as a function of storage temperature. For zeolite-based stabilizers, a modified model incorporating NO_x_ adsorption equilibria is needed, representing an important modeling task not yet addressed in the published literature.

### 5.5. Mechanical Properties and Energetic Plasticizers

For conventional NC propellant grains, mechanical performance can be influenced by molecular-weight characteristics, plasticizer content, and additive chemistry; these relationships should not be transferred directly to NC-containing nanothermite films without matched experimental evidence. Energetic plasticizers such as Bu-NENA [94], glycidyl azide polymer (GAP) derivatives [95], and DEGDN serve dual functions: they lower the glass transition temperature (T_g_) of the NC matrix (enabling flexibility at low temperatures) and contribute additional energetic output. DEGDN-plasticized NC shows T_g_ reductions of 20–40 °C relative to dry NC (T_g_ ~70–100 °C), enabling operational flexibility at temperatures as low as −50 °C [96]. This T_g_ reduction, while beneficial mechanically, also accelerates molecular mobility within the NC matrix and can increase the diffusion rate of stabilizers, NO_x_, and moisture—effects that must be balanced against the mechanical benefits.

Stabilizers and plasticizers primarily address storage stability and mechanical integrity, but advanced ignition systems demand concurrent enhancement of dynamic performance. Section 6 therefore considers NC-containing nanothermite MICs as formulations designed to modify ignition response and energy-release behavior. Their effects on apparent kinetic parameters are formulation- and method-dependent and should not be assumed to follow a universal direction.

## 6. NC–Nanothermite Metastable Intermolecular Composites

In this section, we focus on MIC formulations and architectures that either directly incorporate NC as a binder or can be realistically integrated into NC energetic films. While the broader MIC literature on bare Al/oxide systems is extensive, our emphasis is on how NC’s film-forming, gas-generating, and kinetic properties modify MIC behavior and enable MEMS-compatible deposition. Where bare MIC performance metrics are cited, they serve primarily to contextualize the impact of embedding these systems within NC matrices.

### 6.1. MIC Concept and Thermodynamic Fundamentals

Metastable intermolecular composites (MICs) are physically mixed or nanostructured fuel–oxidizer systems in which at least one component is at the nanoscale, achieving interfacial areas orders of magnitude larger than micron-scale mixtures. The driving force for the thermite reaction (e.g., Al + CuO → Al_2_O_3_ + Cu, ΔH ≈ −4.1 MJ·kg^−1^) is thermodynamically large, but the kinetics in micron-scale mixtures are limited by slow solid-state diffusion [97]. Reducing particle size to 50–200 nm dramatically increases specific interfacial area (from ~0.1 to ~10 m^2^·g^−1^ or greater) [98], enabling reaction timescales of microseconds to milliseconds compared to seconds for micron-scale thermites.

In nanothermite MICs, NC functions as a polymeric energetic binder, providing a continuous organic matrix for dispersing metal and oxide nanoparticles, enabling solution-based deposition and patterning, and coupling its own exothermic denitration to the ignition of the inorganic thermite. At the same time, NC typically represents only a minor mass fraction (often below ~10 wt%) so that the overall energy release and stoichiometry are dominated by the metal/oxide components. Thus, throughout this section, MIC systems are considered as modifiers of NC’s decomposition kinetics and ignition behavior, rather than as stand-alone thermites; NC remains the polymeric energetic material that binds, energizes, and interfaces these MICs with device-scale architectures.

### 6.2. Binary MIC Systems: Al/CuO, Al/Fe_2_O_3_, Al/Bi_2_O_3_, Al/MoO_3_

Al/CuO is one of the most extensively investigated MIC systems. For the superhydrophobic core–shell CuO-Al nanothermites reported by Ke et al., the total heat release measured by DSC reaches about 2.0–2.3 kJ·g^−1^ (fuel-lean compositions with Al/Cu atomic ratios of 0.435 and 0.587), and up to 88% of this energy is retained after one month of storage in air. The functionalized CuO nanotube-Al MICs exhibit steady combustion with flame propagation velocities on the order of 90–105 m·s^−1^ under the tested weak confinement conditions [99]. In NC-based Al/CuO composites, the polymeric matrix modulates the reaction by controlling Al-CuO contact distances and providing supplementary gaseous environment. In one electrospray deposition study, Al/CuO@NC deposits on SCB chips formed fractal-like three-dimensional nanostructures with high surface area, enabling ignition delays as low as 2.8 µs and combustion temperatures estimated at ~4600 °C by optical pyrometry [12] (p. 14).

Al/Fe_2_O_3_ offers a lower reaction enthalpy (~3.9 MJ·kg^−1^) but produces Fe and Fe_3_O_4_ products with favorable mechanical properties for certain applications. In NC matrices, Al/Fe_2_O_3_ MICs exhibit somewhat slower burn rates than Al/CuO analogues but greater compatibility with some binder chemistries. Recent work on Al/Fe_2_O_3_ + RDX nanocomposites [100] intended to replace lead azide in surface detonators demonstrates that nanothermite content can be used to tune initiation sensitivity while controlling detonation performance.

Al/Bi_2_O_3_ produces gaseous Bi vapor at reaction temperatures, providing an additional gas-generation mechanism that enhances pressure output relevant to detonator and EFI applications [101]. Burn rates for Al/Bi_2_O_3_ MICs exceed those of Al/CuO in thin-film geometries, making them attractive for high-speed MEMS initiation despite the higher cost and toxicity of bismuth compounds. Integration of Al/Bi_2_O_3_ MICs with NC matrices—for example in NC-coated Al/Bi_2_O_3_ nanothermites—has been reported but remains substantially less characterized than CuO-based analogues, particularly with respect to long-term aging and ignition variability [102].

Al/MoO_3_ is notable for its high burn rate (up to several km·s^−1^ in multilayer reactive films) and for the volatility of MoO_3_, which creates a reactive vapor-phase oxidizer component supplementing solid-state thermite kinetics. Al/MoO_3_ multilayer films deposited by physical vapor deposition (PVD) onto SCBs achieve highly reproducible ignition performance [103]; their integration with NC binder layers has been demonstrated as a route to combining the gas-generating and binding properties of NC with the high burn rate of Al/MoO_3_.

For MEMS-SCB integration, Al/CuO remains the most mature system owing to its well-characterized ignition threshold and compatibility with electrospray deposition, but Al/Bi_2_O_3_ offers a significant gas-generation advantage relevant to pressure-driven output devices—an advantage that has not been systematically compared against Al/CuO under identical bridge energies. Al/MoO_3_ multilayer films deposited by PVD onto SCBs achieve highly reproducible ignition performance, but the vacuum deposition route is incompatible with solution-processed NC binders, fundamentally limiting direct co-formulation in a single continuous NC–MIC film. In NC-based architectures, Al/MoO_3_ therefore functions primarily as a separate reactive multilayer cap that is coupled to an underlying NC-based energetic layer via thermal and gas-phase coupling, rather than as a component of a single, co-deposited NC–MIC. From the NC perspective, Bi_2_O_3_-containing MICs illustrate how the polymeric matrix can be leveraged to manage gas generation and confinement: NC’s film-forming ability and tunable porosity allow control over Bi vapor release pathways, which in turn affects pressure-time profiles and autocatalytic NO_x_ dynamics in NC/Bi_2_O_3_ composites.

Beyond classical Al/oxide nanothermites, NC also functions as a polymeric energetic binder in AN/Mg-based heterogeneous composites [104], where its interaction with catalytic additives can be exploited to tailor decomposition behavior. Activated carbon supported nanosized cobalt oxide (AC-Co_3_O_4_) [105] has been shown to substantially improve the thermal characteristics of AN/Mg/NC formulations, decreasing peak decomposition temperatures and enhancing overall combustion efficiency relative to the parent AN/Mg/NC system. These results highlight that NC-containing AN/Mg composites can be tuned not only by varying NC loading, but also by engineering catalytic interfaces through supported transition metal oxides.

These AN/Mg/NC case studies illustrate that the thermal and ignition behavior of heterogeneous energetic composites depends on formulation composition, additive chemistry, particle dispersion, and interfacial architecture. The independent roles of NC nitrogen content, molecular-weight characteristics, crystallinity, and morphology require matched studies before general conclusions can be drawn.

### 6.3. Ternary Systems: MgAl–M_x_O_y_ Alloy Nanothermites

Ternary metastable intermolecular composites based on MgAl alloy fuel particles and metal–oxide oxidizers, including CuO, NiO, and TiO_2_, have been investigated as high-reactivity additives for NC-containing energetic films [106]. Arrested reactive milling can produce refined MgAl–oxide particles with intimate fuel–oxidizer contact, which may improve ignition behavior relative to physically mixed systems.

In the MgAl–CuO/NC formulation reported in [107], the mean apparent activation energy obtained by the stated kinetic procedure was approximately 163 kJ mol^−1^, compared with approximately 154 kJ mol^−1^ for the pure-NC reference measured in that study. Despite this increase in the reported apparent E_a_, the ignition delay on a microbridge decreased from approximately 100 to 51 ms. This result demonstrates that apparent activation energy obtained from bulk thermal analysis must not be interpreted as a universal predictor of device ignition delay. In particular, interfacial heat release, local heat-transfer pathways, particle dispersion, electrical energy coupling, and gas generation can accelerate ignition even when the fitted apparent E_a_ is unchanged or higher.

Results for MgAl–NiO/NC and MgAl–TiO_2_/NC should likewise be reported only according to the kinetic method, reference material, formulation composition, and test conditions used in the original study. Cross-formulation statements that MgAl-based nanothermites systematically lower E_a_ should be avoided unless the compared values were obtained by the same method using matched samples and heating conditions. The available evidence more securely supports the conclusion that these additives can modify ignition response, rather than a universal direction of change in apparent activation energy.

### 6.4. Core–Shell and Reactive Multilayer Film Architectures

Core–shell nanothermites—in which a fuel core (e.g., Al) is conformally coated with a thin oxide shell (e.g., CuO, Fe_2_O_3_) by atomic layer deposition (ALD) or solution precipitation—offer superior contact between fuel and oxidizer at the molecular scale, reducing the diffusion distance for oxygen transfer to near-zero and dramatically increasing reactivity. In NC matrices, core–shell particles provide the same binding and gas-generating benefits as simple MICs, but with more predictable stoichiometry and higher batch-to-batch consistency. ALD-coated Al@CuO nanoparticles in NC films have been reported to reduce ignition temperatures by 30–50 °C relative to physically mixed Al/CuO/NC composites [108].

Reactive multilayer films (RMLs)—periodic nanometer-scale laminates of fuel and oxidizer layers deposited by magnetron sputtering or PVD—represent an architecturally different approach to nanoenergetics that is highly compatible with MEMS fabrication. Al/CuO, Al/MoO_3_, and Al/Ni RMLs with bilayer thicknesses of 10–100 nm exhibit self-propagating reactions with velocities of 1–10 m·s^−1^ and heats of reaction of 1–4 MJ·kg^−1^, adjustable by controlling bilayer period. Integration of RML caps with NC-based ignition layers provides a hybrid architecture where NC triggers RML ignition at low electrical energy input, and the RML then delivers a high-energy thermal pulse to secondary charges [109]. This hierarchical design approach has significant potential for miniaturized all-integrated energetic chips but requires careful interface engineering to prevent interdiffusion during deposition.

### 6.5. Kinetic and Ignition Modulation in NC–MIC Formulations

Nanothermite additives can alter the apparent thermal-kinetic parameters and ignition behavior of NC-containing formulations through chemical interactions and heat- and mass-transfer effects. However, the direction and magnitude of any reported change in apparent E_a_ depend on the formulation, kinetic model, heating program, sample geometry, and reference material [110]. Consequently, apparent activation energies from different studies or kinetic methods should not be compared as if they were universal intrinsic constants of the NC–MIC system.

Metal–oxide surfaces may interact with nitrate-ester groups, modify local NO_x_ chemistry, or alter the local thermal environment during heating [111]. At the device scale, particle–particle contact, interfacial heat release, hot-spot formation, film porosity, electrical coupling, and gas transport can change ignition delay independently of the apparent E_a_ obtained from bulk DSC or TGA analysis. Distinguishing chemical effects from transport and device-scale effects requires matched formulation series and consistent kinetic and ignition protocols.

### 6.6. Structure–Activity Relationships: Particle Size, Dispersion, Interface Quality

Performance of NC-based MICs is critically dependent on nanoparticle dispersion quality. Well-dispersed particles with narrow size distributions and minimal agglomeration maximize fuel–oxidizer contact area and produce homogeneous, thin-film-compatible composites. SEM analysis of optimally processed MgAl–CuO/NC films reveals uniformly distributed nanodomains of 50–200 nm embedded in the NC matrix, with porosity facilitating gas transport [112]. In contrast, poorly dispersed agglomerates (>1 µm) act as inert fillers that physically disrupt the NC matrix without providing catalytic or reactive benefits, sometimes actually degrading ignition performance.

The passivation layer on aluminum nanoparticles (typically γ-Al_2_O_3_, 2–5 nm thick) presents a barrier to oxide–fuel contact and a variable that is frequently not well characterized or controlled in published studies. Al_2_O_3_ shell thickness, structure (amorphous vs. γ-Al_2_O_3_ vs. α-Al_2_O_3_), and coverage completeness all affect the temperature and rate of Al oxidation onset, contributing to irreproducibility between batches and laboratories [113]. This variable requires routine characterization (e.g., TEM, XPS) as a standard part of MIC reporting.

### 6.7. Sensitivity Characterization of NC–Nanothermite Composites

The high reactivity of NC–nanothermite MICs that makes them attractive for MEMS ignition applications simultaneously elevates their sensitivity to accidental stimuli. Uncoated Al/CuO nanothermite powders exhibit a minimum ESD ignition energy as low as 25 mJ—above the human body static discharge—and friction sensitivity well below the 80 N threshold that defines primary explosive-level hazard. These properties necessitate careful assessment before any MEMS integration process [114].

NC binder loading plays a direct role in modulating sensitivity. Al/CuO composites containing 2.5 wt% carbon nanosheet (CN, structurally analogous to NC in its insulating function) achieve an ESD ignition threshold of 3.2 mJ, rising to 8.45 mJ at 10 wt% loading. NC-coated Al/Bi_2_O_3_ nanothermites similarly demonstrate improved ESD safety relative to uncoated analogues while maintaining high energy output. The mechanism is primarily electrical: NC and carbon-based coatings reduce inter-particle conductivity, suppressing the joule heating pathway responsible for ESD ignition in Al/CuO [115].

Despite this relevance, systematic Federal Institute for Materials Research and Testing (Bundesanstalt für Materialforschung, BAM) impact and friction sensitivity data for NC–nanothermite composites as a function of NC loading, particle size, and film morphology are scarce. Most available sensitivity data derive from loose powder measurements, which do not represent the thin-film geometry of MEMS-deposited energetic layers. Whether electrospray-deposited Al/CuO@NC films on SCB chips exhibit the same ESD thresholds as bulk powder composites remains uncharacterized, as thin-film-specific ESD and BAM sensitivity data are essentially absent—a significant gap given that CMOS firing circuits operate in environments where static discharge is a realistic hazard. Standardized testing per MIL-STD-1751A [116] applied specifically to MEMS-format thin films represents a critical unmet need in the field.

### 6.8. Safety–Performance Trade-Offs in NC Formulations

Reported NC-containing energetic formulations involve practical trade-offs among ignition response, thermal stability, mechanical integrity, processability, and sensitivity. Because the magnitude and even direction of these effects depend on formulation composition, additive loading, deposition route, sample geometry, and test protocol, they should not be interpreted as universal rules. Table 5 summarizes formulation-specific considerations and the principal limitations of the currently available evidence.

For on-chip MEMS devices, rapid ignition must be balanced against reproducible long-term storage stability and acceptable safety margins. However, this balance cannot be inferred from a single apparent activation energy or from an individual NC structural descriptor. It must be assessed for the complete energetic film through matched thermal, aging, sensitivity, and device-level ignition measurements.

A potentially useful formulation strategy is to combine stabilizing and ignition-promoting functions spatially or compositionally within a device; for example, a stabilized NC-containing matrix may be used together with localized nanothermite regions. Such approaches remain formulation-specific hypotheses and require experimental validation under realistic device conditions, including thermal cycling, humidity exposure, electrostatic-discharge testing, and statistical ignition measurements.

These considerations directly affect micro-ignition architectures, in which bridge geometry, electrical energy delivery, film thickness, particle dispersion, and energetic-layer design jointly determine ignition reliability. Section 7 therefore examines SCB, EFI, and MEMS igniter configurations, focusing on the reported performance and remaining qualification gaps of NC-containing energetic films. Section 7 therefore integrates the preceding materials-level insights into concrete SCB, EFI, and MEMS igniter designs, focusing on how NC-based MIC films perform under realistic device constraints.

### 6.9. Functional Qualification Criteria for NC in Nanothermite-Containing Films

Rather than prescribing universal thresholds for nitrogen content, molecular weight, crystallinity, or morphology, NC should be qualified for a specific nanothermite formulation according to its demonstrated functional performance in the intended process and device architecture. The following criteria are proposed as a practical reporting and qualification framework:

**Film formation and adhesion.** The NC-containing formulation should form a continuous and adherent layer on the intended substrate after the selected deposition process, without cracking, delamination, or unacceptable residual solvent.

**Nanoparticle dispersion.** The NC matrix should enable reproducible dispersion of fuel and oxidizer particles at the selected solids loading. SEM or comparable characterization should document agglomeration, film uniformity, and interfacial contact.

**Process compatibility.** The solvent system, drying conditions, and deposition route should be compatible with the substrate, device materials, and fabrication sequence. NC deposition should be positioned after high-temperature processing steps when required by its thermal sensitivity.

**Thermal and ignition response.** The complete NC–MIC formulation—not NC alone—should be evaluated by appropriate thermal analysis and, where relevant, device-level ignition testing. Reported values should include the test method, heating rate or electrical input, sample geometry, and replicate number.

**Storage and safety assessment.** Stability under relevant temperature and humidity conditions, as well as ESD, friction, and impact sensitivity where applicable, should be evaluated for the final formulation and thin-film geometry. Bulk-powder values should not be assumed to represent deposited device films.

**Batch traceability.** NC grade, nitrogen content, solvent, stabilizer, source, and available molecular-weight or crystallinity data should be recorded for reproducibility. These descriptors are quality-control information; current evidence does not justify universal acceptance limits for them in nanothermite applications.

## 7. Integration in Micro-Ignition Devices

### 7.1. Semiconductor Bridge (SCB) Igniters

Semiconductor bridge (SCB) initiators use a thin polysilicon bridge element that, under typical capacitor-discharge conditions, reaches temperatures of several thousand Kelvin within microseconds, creating a high-temperature plasma and shock wave that ignites the adjacent energetic layer [117]. Key performance metrics include all-fire energy (minimum firing energy, MFE, typically 1–100 µJ), no-fire energy (maximum safe current that does not cause ignition), ignition delay, and output pressure.

NC-based energetic films deposited on SCBs provide both improved adhesion relative to inorganic energetic layers and supplementary gaseous products that enhance output. When NC is used as the matrix for Al/CuO nanothermite (electrospray-deposited Al/CuO@NC on SCB) [12] (p. 14), ignition delays as low as 2.8 µs are achieved—compared to ~200 µs for pure NC films and ~50 µs for NC + DPA films under comparable electrical inputs. The output of these composite devices reliably ignites secondary B/KNO_3_ propellant charges, demonstrating cascaded ignition capability suitable for miniaturized pyrotechnic trains.

**Figure 5:**
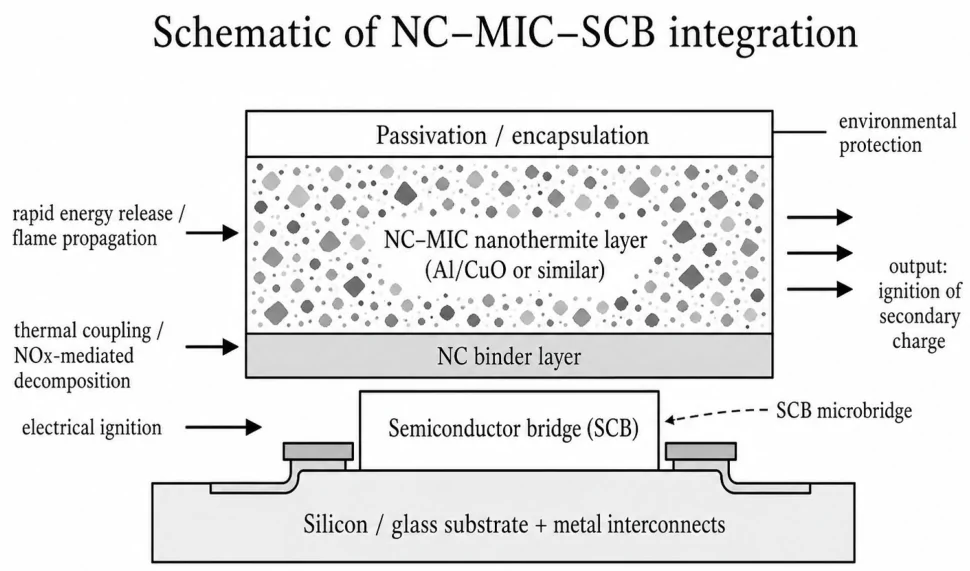
Schematic of NC–MIC–SCB integration (layered device schematic). Layer thicknesses and bridge dimensions are representative of reported SCB micro-igniters (bridge length 100–300 µm, NC–MIC film thickness 5–20 µm).

Schematic cross-section of a semiconductor bridge micro-igniter incorporating NC-based energetic films: NC or NC–MIC layers are deposited on the polysilicon bridge, overlying secondary energetic charges and encapsulation. This illustration, presented in Figure 5, highlights NC’s role as a polymeric energetic material in device integration.

It is important to distinguish between ignition delays achieved via resistive (Ohmic) heating—where pure NC films on Ni-Cr micro-bridges ignite in ~30–100 ms dominated by thermal conduction and autocatalytic induction [118]—and those achieved via semiconductor bridge (SCB) plasma discharge, where ignition delays for pure NC fall to ~200 µs and for NC–nanothermite composites to as low as 2.8 µs [9] (p. 2), owing to convective rather than conductive energy transfer.

### 7.2. Exploding Foil Initiators (EFIs)

Exploding foil initiators (EFIs) [119,120,121] use a thin metallic foil (typically Cu or Al, ~0.1–1 µm thick) that is vaporized by a capacitor-discharge current pulse, generating a high-velocity plasma jet that impacts and shock-initiates a secondary explosive. NC-based energetic layers in EFI architectures serve primarily as adhesive and sensitivity-enhancing interfaces between the foil and the secondary charge. The thin-film processability of NC enables conformal coating of complex EFI geometries, while the addition of nanothermite components increases shock sensitivity to levels compatible with less shock-sensitive secondary explosives, potentially allowing the use of insensitive munition (IM) candidates in EFI-initiated systems.

### 7.3. MEMS Micro-Igniters

MEMS-fabricated igniters exploit photolithography, deep reactive ion etching (DRIE), and thin-film deposition to create fully miniaturized ignition devices with bridge resistances of 1–100 Ω, energy inputs in the range of 0.1–10 mJ, and dimensions compatible with chip-scale pyrotechnic systems [122,123,124]. NC-based energetic films are attractive for MEMS integration because of their solvent processability: NC dissolved in acetone or ethyl acetate can be deposited by spin-coating, dip-coating, inkjet printing, or electrospray, avoiding the high-vacuum deposition equipment required for PVD-based reactive films [125,126,127]. MEMS safe-arm-and-fire (SAF) devices incorporating NC-based charges have been demonstrated with all-fire energies below 1 mJ and reliable operations across the temperature ranges (e.g., −54 °C to +74 °C per MIL-STD-810), consistent with military environmental standards.

A critical but underreported challenge in MEMS-scale NC devices is the statistical variability of ignition performance across arrays of nominally identical chips. Deposition inhomogeneities at the nanometer scale—thickness variations, porosity differences, particle size distributions in MICs—translate into chip-to-chip variability in MFE and ignition delay. For safety-critical applications, the distribution tails (not just mean values) must be characterized, yet the majority of published MEMS igniter studies report only mean ignition delays without distributional statistics.

### 7.4. Additive Manufacturing of Energetic Films

Additive manufacturing techniques—particularly direct ink writing (DIW), inkjet printing, and aerosol jet printing—have emerged as transformative tools for fabricating structured energetic layers on MEMS substrates [128]. NC functions as an excellent ink vehicle: dissolved at 5–30 wt% in acetone or ethyl acetate with nanothermite particles suspended at up to 97 wt% total solids loading (in NC binder), NC-based MIC inks support DIW printing of energetic lines with widths of 100–1000 µm and consistent combustion characteristics. Spray-dried nAl/CuO/NC microspheres, used as the solid component of DIW inks, produce printed energetic lines with higher explosion heats and faster heating rates than physically mixed Al/CuO analogues, and successfully ignite Ni–Cr bridge ignition devices [129], demonstrating direct compatibility with standard MEMS bridge architecture.

Inkjet printing of NC-based energetic inks onto pre-patterned MEMS structures offers the additional advantage of maskless, digital patterning with droplet volumes of 1–100 pL, enabling sub-100-µm feature resolution and localized deposition of different formulations within a single device [130]. A novel approach combining DIW and physical vapor deposition (PVD) has demonstrated fast-response, low-energy-input micro-igniters with Al/Ni reactive multilayer caps on NC-based energetic pads, achieving ignition with sub-mJ electrical inputs [131]. Challenges include ink rheology control (requiring precise NC concentration and nanothermite loading for stable jetting without clogging or satellite droplets), solvent management (avoiding NC precipitation during drying), and post-deposition residual stress management in films on silicon substrates.

### 7.5. CMOS Compatibility and Process Integration

Integration of NC-based energetic films with CMOS electronics requires careful management of process temperatures, chemical compatibility, and contamination risks [132]. NC decomposes at temperatures above approximately 180–200 °C, which is below the standard CMOS back-end-of-line (BEOL) processing temperatures (~400 °C) [133], meaning that energetic layer deposition must be the final step in the fabrication sequence. Solvent compatibility with CMOS dielectrics (SiO_2_, Si_3_N_4_) and metal interconnects (Al, Cu) must be verified; acetone and ethyl acetate are generally compatible with standard CMOS materials if contact times are controlled [134]. Energetic film patterning by laser micromachining (rather than photolithography, which may expose NC to developer solvents and UV radiation) represents a practical approach.

### 7.6. Reliability, Aging, and Statistical Ignition Variability

Long-term reliability assessment of NC-based thin-film devices must ultimately be benchmarked against established qualification frameworks. For conventional NC propellant systems, AOP-48 (Edition 2)—which superseded STANAG 4527 [135] and consolidates NATO standardized protocols [136] for stabilizer depletion measurement and chemical life prediction—provides the reference methodology: accelerated aging at 70 °C for 35 days is used to simulate ~10 years of storage at 25 °C, based on an assumed E_a_ of 120 kJ·mol^−1^ above 60 °C. However, no equivalent standardized protocol currently exists for MEMS-integrated NC thin films, where film geometry, substrate thermal conductivity, and the absence of grain confinement fundamentally alter the aging kinetics relative to bulk propellant grains. Adaptation of AOP-48 methodology to chip-scale energetic devices represents a critical regulatory and metrological gap.

Taken together, current evidence shows that NC remains a uniquely versatile polymeric energetic material for micro-ignition systems, but its autocatalytic NO_x_-driven instability and complex E_a_(α) landscape demand more sophisticated formulation and testing strategies. Zeolite-type porous stabilizers and MgAl–nanothermite MICs offer powerful, complementary routes to modulate stability and ignition thresholds, yet their long-term behavior in MEMS-integrated thin films and their statistical safety margins are still poorly quantified. Progress will depend on harmonized kinetic and aging protocols, operando and spatially resolved diagnostics, and systematic sensitivity and variability studies at device scale, enabling rational NC-based energetic film design that balances shelf-life, safety, and ultrafast ignition performance.

To address this gap, future work should adapt AOP-48 stability frameworks to thin-film geometries by combining accelerated aging of NC-based MEMS devices with high-throughput ignition statistics across large arrays (N ≫ 100) of nominally identical chips. Built-in self-test concepts developed for MEMS sensors and actuators could be leveraged to monitor on-chip resistance drift, leakage currents, and partial discharge events as proxies for energetic layer degradation. Coupling such electrical diagnostics with periodic UHPLC or micro-FTIR analysis of stabilizer depletion in situ would provide the data needed to formulate chip-specific life-prediction models analogous to bulk propellant standards.

The absence of standardized reliability protocols and operando diagnostics for MEMS-integrated NC films motivates the broader research gap analysis in Section 8. There, we synthesize methodological, scaling, and aging challenges into a structured agenda that ultimately informs the roadmap and future directions outlined in Section 9.

## 8. Critical Analysis and Research Gaps

### 8.1. Methodological Limitations in Kinetic Studies

The wide dispersion of reported E_a_ values for NC (154 kJ·mol^−1^ for “pure” NC) reflects not only genuine sample variability but also methodological inconsistency: different DSC instruments, sample masses (0.5–5 mg), crucible types (open vs. sealed), heating rate ranges (1–20 K·min^−1^), and kinetic software implementations all introduce systematic biases. The kinetic compensation effect further obscures genuine compositional differences. The field would benefit substantially from an international round-robin study analogous to those conducted by ICTAC for other polymer systems, establishing reference values for well-characterized NC standards and enabling reliable cross-laboratory comparison.

### 8.2. Scale-Up Gap: Milligram DSC to Device-Scale Ignition

A fundamental disconnect separates milligram-scale DSC/TGA experiments and actual device-scale ignition dynamics in SCB or EFI architectures. At the device scale, heat transfer from the bridge into the energetic film, heat sinking into the silicon substrate, NO_x_ transport in confined microvolumes, and mechanical constraints (confinement pressure) all modify ignition dynamics in ways not captured by bulk TGA measurements. Bridge current density, pulse duration, and film porosity create spatial temperature gradients within the energetic layer that are inconsistent with the spatially uniform heating assumed in DSC kinetics. Finite-element models coupling electrical, thermal, and chemical kinetic equations are needed—and partially available—but require accurate temperature-dependent kinetic inputs in the form of E_a_(α) profiles rather than single-value Arrhenius parameters. Current practice—inserting a single Kissinger-derived E_a_ into device-level thermal models—systematically underestimates ignition delay at low bridge energies and overestimates it at high energies, because the conversion-dependent acceleration of NC decomposition is not represented.

Isoconversional E_a_(α) data from KAS or advanced Vyazovkin analysis, combined with a reaction model f(α) identified by master-plot methods, would provide the minimal kinetic dataset needed for predictive finite-element simulation of SCB ignition. A further complication arises from the nanothermite component: the aluminum (Al) oxidation kinetics, which govern the exothermic thermite reaction at the bridge–film interface, are strongly dependent on Al particle size, passivation layer thickness, and local stoichiometry—parameters that vary between batches and are rarely reported with sufficient detail to enable quantitative modeling. Bridging the milligram-to-device scale gap therefore requires a coordinated effort combining (i) standardized isoconversional kinetic characterization of NC-MIC films at multiple heating rates; (ii) spatially resolved temperature measurements during SCB firing using embedded resistance thermometry or transient thermoreflectance; and (iii) validated finite-element models that couple Joule heating, heat conduction into the substrate, and NC/MIC reaction kinetics with conversion-dependent E_a_(α). Until this infrastructure is established, device performance predictions will remain semi-empirical, limiting the rational optimization of firing energy, pulse duration, and film composition for next-generation miniaturized initiators.

### 8.3. Long-Term Stability: Absent Data for MEMS-Integrated NC Films

Perhaps the most practically consequential research gap identified in this review is the near-complete absence of systematic long-term aging data for NC and NC–nanothermite films in MEMS-integrated configurations. The overwhelming majority of published ignition performance studies characterize freshly prepared devices tested within days or weeks of fabrication. Real applications—munitions, aerospace pyrotechnics, automotive safety systems—require demonstrated reliability over storage periods of 10–25 years under variable temperature and humidity conditions. For bulk NC propellants, service-life prediction protocols based on stabilizer consumption kinetics and VST measurements are well established. However, no equivalent framework has been demonstrated for MEMS-scale NC films, where geometric confinement, substrate interactions, and nanothermite particle dynamics introduce fundamentally different aging mechanisms.

Accelerated aging studies at 60–80 °C with periodic measurement of E_a_ (by isoconversional DSC), stabilizer content (by HPLC or FTIR), and ignition delay (on representative chips) would provide the minimal dataset needed to develop service-life models. The challenge of characterizing nanogram-to-microgram quantities of energetic material on a chip—well below the sensitivity threshold of conventional TGA instruments—may require specialized calorimetric techniques such as nanocalorimetry or chip-scale differential scanning calorimetry, which represents a priority direction where instrument development and materials science must co-evolve.

### 8.4. Operando Diagnostics: Needs and Challenges

Mechanistic understanding of NC and NC–nanothermite decomposition would be substantially advanced by operando (in situ, real-time) diagnostics applied during heating, decomposition, and ignition events. Currently, the mechanistic picture is assembled largely from post-mortem analysis (SEM/TEM of residues, FTIR of collected gases) and bulk calorimetric measurements that average over heterogeneous processes. High-speed optical diagnostics—streak cameras, ICCD imaging, CARS thermometry—have been applied to macroscopic MIC combustion, but their application to MEMS-scale events, where the entire reaction zone is ~100 µm and the reaction duration is ~1 µs, poses severe technical challenges. Synchrotron-based time-resolved X-ray diffraction during rapid heating (T-jump/SXRD) has been demonstrated for energetic crystals and could in principle be adapted to NC composite thin films, enabling direct observation of crystalline phase changes in nanothermite components during reaction. Similarly, embedded micro-thermocouples or MEMS-integrated resistance thermometers could provide temperature histories during ignition events, validating device-scale thermal models. Investment in these diagnostic capabilities is essential for moving the field from empirical performance characterization to mechanistically grounded device design.

## 9. Conclusions

This review shows that nitrocellulose remains relevant in selected nanothermite-containing energetic films primarily because it serves as a solution-processable binder, film-forming matrix, dispersing medium, and ignition-coupling component. In typical NC–nanothermite formulations, NC is a minor mass fraction, whereas the metal/oxide components dominate the overall thermochemical output. NC should therefore not be described as a universal platform for nanothermites.

The available evidence indicates that formulation-specific factors—including NC loading, stabilizer selection, nanothermite composition, particle dispersion, deposition route, film architecture, and electrical ignition conditions—are the most useful variables for interpreting reported thermal and ignition behavior. Apparent activation energies obtained by different kinetic methods, sample geometries, and heating programs should not be compared directly or used alone to predict device ignition response. Current evidence also does not support universal requirements for NC nitrogen content, molecular-weight distribution, crystallinity, or morphology in nanothermite applications.

For practical formulation development, NC should be qualified by functional criteria: film continuity and adhesion, nanoparticle dispersion, process compatibility, reproducible thermal and ignition response, storage stability, safety, and batch traceability. Future work should combine NC characterization with matched thermal, aging, sensitivity, and device-level measurements to establish formulation-specific structure–performance relationships.

## Figures and Tables

**Table 1:** Comparative properties of NC and principal alternative energetic polymers relevant to MEMS ignition applications, highlighting trade-offs among energetic functionality, thermal stability, film-forming behavior, solvent processability, and reported CMOS/MEMS compatibility. Representative values are compiled from the literature cited in Section “Nitrocellulose as a Polymeric Energetic Material: Scope and Rationale” and are intended for qualitative comparison.

Property	NC (%N)	GAP	polyNIMMO	polyGLYN
Energetic group	-O-NO_2_	-N_3_	-O-NO_2_	-O-NO_2_
%N (wt%)	13.5	~18	~9.5	~12.7
T_g_ (°C) *	~55 (plasticized film; dry NC: 70–100 °C)	−45	−25	−35
T_d_ onset	~170–190	~242	>200	~200
ΔH_d_ (kJ/g)	~2.0–3.0	~2.0	~1.5	~2.3
Film-forming	Excellent	Requires crosslinking	Requires crosslinking	Requires crosslinking
Solvent processability	Acetone, esters	Limited	Limited	Limited
MEMS/CMOS compatibility	Demonstrated	Not reported	Not reported	Not reported
Thermal stability (storage)	Moderate (autocatalytic)	High	Moderate	Moderate
Cost/maturity	Low/very high	Moderate/high	High/moderate	High/lower
Nanothermite binder use	Extensively reported	GAP-ETPE/MIC reported	Limited	Limited

* Reported dry-NC glass-transition values depend on NC grade, degree of substitution, plasticizer content, moisture, and measurement conditions.

**Table 2:** Classification of NC by nitrogen content and primary applications.

Nitrogen Content (%N)	Grade	Primary Applications	References
10.7–11.2	Collodion/lacquer grade	Varnishes, inks, adhesives, membranes	[22,23,24]
11.3–12.2	Pyroxylin	Celluloid, plastics, coatings	[25,26,27]
12.2–12.6	Smokeless powder grade	Single/double-base propellants	[28,29,30]
>12.6	Guncotton/explosive grade	Explosives, MICs, SCB initiators	[31,32,33]

**Table 3:** Overview of kinetic methods applied to nitrocellulose thermal decomposition.

Method	Equation Form	Key Assumption	Strengths for NC	Limitations for NC
Kissinger	Peak-based ln(β/Tp^2^) vs. 1/Tp	Single dominant step	Rapid comparison of formulations	Mechanistically inadequate for autocatalytic multi-step behavior
OFW	lnβ vs. 1/Tα at fixed α	Doyle approximation	Simple, widely used integral method	Sensitive to heating-rate range; overestimates E_a_ at high α
KAS	ln(β/Tα^2^) vs. 1/Tα	Improved integral approximation	Better accuracy than OFW for polymers	Still model-free; does not resolve mechanism explicitly
Friedman	ln(dα/dt) vs. 1/Tα	Differential form, no integral approximation	High sensitivity to multi-step regimes	Amplifies experimental noise; requires high-quality data
Advanced Vyazovkin	Minimization of exact integral functional	Model-free with exact integral	Most accurate E_a_(α) for NC composites	Computationally intensive, less commonly implement

**Table 4:** Apparent activation energies (E_a_) for NC-based systems determined by various kinetic methods.

System	Method	E_a_ (kJ/mol)	T_peak_ (°C)	References
Pure NC (~ 12.6%N, thin film)	OFW	~154	195–212	[74]
NC + DPA (2 wt%)	OFW	168–201	200–218	[75]
MnO_2_/Al/NC	OFW	~162	~188	[76]
NC + DPA (1 wt%)	KAS	147–170	200–215	[75]
NC + Cr_2_O_3_ nanoparticles	KAS	140–158	185–195	[77]
NC + DEGDN +AC-CoO	KAS	~99.2	~185	[78]
NC + Al/CuO (electrospray)	KAS	130–145	180–190	[79]
NC + lignin stabilizer	Friedman	155–182	200–210	[80]
NC + MgAl-CuO (5 wt%)	Friedman	~163	185–195	[81]
NC + Cu-ZSM-5 (3 wt%)	Advanced Vyazovkin	174–196	205–220	[72]
NC + Ag-ZSM-5 (5 wt%)	Advanced Vyazovkin	168–188	203–218	[73]

Note: Apparent activation energies in this table were obtained using different kinetic methods, heating-rate programs, sample forms, and formulations. They are therefore not directly comparable across studies and should not be interpreted as universal indicators of ignition performance. Comparisons are most meaningful only within a matched study using the same kinetic method and experimental protocol.

**Table 5:** Formulation-specific safety and performance considerations reported for selected NC-containing energetic systems.

Modification or Formulation Factor	Possible Performance Outcome in Reported Formulations	Evidence Limitation
Nitrogen content within a controlled NC series	May alter decomposition temperature and heat release	Not established as a universal NC–MIC rule
Nanothermite addition	May reduce ignition delay or threshold in selected formulations	Depends on composition, loading, dispersion, film geometry, and ignition method
Energetic plasticizer	May increase flexibility and energetic output	Migration and stability effects are formulation-specific
Stabilizer or zeolite addition	May improve thermal stability in reported NC systems	Cross-study ranking requires matched aging and kinetic protocols
Film architecture	May influence deposition quality, gas transport, and device ignition behavior	No universal morphology–decomposition relationship is established

## Data Availability

The original contributions presented in this study are included in the article. Further inquiries can be directed to the corresponding author.

## Abbreviations

The following abbreviations are used in this manuscript:

AGU	Anhydroglucose unit
BAM	Bundesanstalt für Materialforschung (Federal Inst. for Materials Research and Testing)
BuNENA	N-butyl-N-(2-nitroxyethyl)nitramine
CARS	Coherent anti-Stokes Raman spectroscopy
CI	Crystallinity index
CMOS	Complementary metal–oxide semiconductor
DAEM	Distributed activation energy model
DEGDN	Diethylene glycol dinitrate
DIW	Direct ink writing
DP	Degree of polymerization
DPA	Diphenylamine
DS	Degree of substitution
DSC	Differential scanning calorimetry
EFI	Exploding foil initiator
ESD	Electrostatic discharge
GAP	Glycidyl azide polymer
GPC	Gel permeation chromatography
ICTAC	International Confederation for Thermal Analysis and Calorimetry
KAS	Kissinger–Akahira–Sunose (method)
KCE	Kinetic compensation effect
MEMS	Micro-electromechanical systems
MIC	Metastable intermolecular composite
NC	Nitrocellulose
OFW	Ozawa–Flynn–Wall (method)
PETN	Pentaerythritol tetranitrate
polyGLYN	Poly(glycidyl nitrate)
polyNIMMO	Poly(3-nitratomethyl-3-methyloxetane)
PVD	Physical vapor deposition
RDX	1,3,5-trinitro-1,3,5-triazinane
RML	Reactive multilayer film
SCB	Semiconductor bridge
SEM	Scanning electron microscopy
SXRD	Synchrotron X-ray Diffraction
TAS	Trache–Abdelaziz–Siwani
TG-FTIR	Thermogravimetric Analysis coupled with Fourier Transform Infrared Spectroscopy
TG-MS	Thermogravimetric Analysis—Mass Spectrometry
TGA	Thermogravimetric analysis
VST	Vacuum stability test
WAXS	Wide-angle X-ray scattering
%N	Nitrogen content (weight percent)
